# Correlation between quantitative assessment of contrast enhancement in contrast-enhanced spectral mammography (CESM) and histopathology—preliminary results

**DOI:** 10.1007/s00330-019-06232-6

**Published:** 2019-05-14

**Authors:** Wojciech Rudnicki, Sylwia Heinze, Joanna Niemiec, Zbigniew Kojs, Beata Sas-Korczynska, Ed Hendrick, Elzbieta Luczynska

**Affiliations:** 1 Department of Radiology, Maria Sklodowska-Curie Memorial Cancer Centre and Institute of Oncology, Cracow, Poland; 2 Department of Systemic and Generalized Malignancies, Maria Sklodowska-Curie Memorial Cancer Centre and Institute of Oncology, Cracow, Poland; 3 Department of Gynecologic Oncology, Maria Sklodowska-Curie Memorial Cancer Centre and Institute of Oncology, Cracow, Poland; 4 Department of Oncology, Maria Sklodowska-Curie Memorial Cancer Centre and Institute of Oncology, Cracow, Poland; 5 Radiology Department, University of Colorado – Denver, School of Medicine, 12700 E 19th Ave., Aurora, CO 80045 USA

**Keywords:** Breast cancer, Breast neoplasms, Digital mammography, Contrast media

## Abstract

**Objectives:**

Contrast-enhanced spectral mammography (CESM) is a novel method for breast cancer detection. The aim of this study is to check if there is a possibility of quantitative assessment of contrast enhancement in CESM and if there is any correlation between quantitative assessment of contrast enhancement in CESM and histopathology.

**Methods:**

A total of 167 female patients underwent CESM. All subjects previously had suspicious lesions found on mammography, breast ultrasound, or both. After imaging, the following parameters were evaluated: number of enhancing lesions in each breast and size and degree of enhancement of each lesion. Based on the collected data, the percentage signal difference between enhancing lesion and background (%RS) and signal-difference-to-noise ratio (SDNR) were measured for each lesion.

**Results:**

The number of lesions detected in the study population was 195. Among all diagnosed lesions, 120 (62%) were assessed to be infiltrating cancers, 16 (8%) non-infiltrating cancers, and 59 (30%) were benign. Thirteen (7%) lesions did not enhance in CESM; all non-enhancing lesions were confirmed to be benign under histopathological examination. Analysis of enhancement indices showed that signal values within lesions and signal values within background ROIs (regions of interest) were similar in CC (craniocaudal) and MLO (mediolateral) projections. Mean %RS values were correlated with the type of enhancing lesion, infiltrating cancers having the highest values, benign lesions the lowest.

**Conclusions:**

This work has demonstrated a significant correlation between the degree of lesion enhancement in CESM and malignancy. Quantitative analysis of enhancement levels in CESM can distinguish between invasive cancers and benign or in situ lesions.

**Key Points:**

*• There is a possibility of quantitative assessment of contrast enhancement in CESM.*

*• Correlation between quantitative assessment of contrast enhancement in CESM and histopathology was observed.*

## Introduction

Contrast-enhanced spectral mammography (CESM) is a novel method for breast cancer detection. This new mammography (MG) technique has been introduced to combine the benefits of mammography (such as low cost, more comfortable patient positioning, short examination time) with those of dynamic contrast-enhanced imaging techniques (revealing malignancy through display of angiogenesis, as occurs in breast magnetic resonance imaging, MRI). CESM is based on dual-energy mammography in which two images are acquired with different x-ray energies, one below the k-edge of an iodinated contrast agent and the other above the k-edge, with the same breast positioning. After intravenous iodine contrast agent administration, low- and high-energy images are obtained: the low-energy conventional MG image acquisition (about 26–32 peak kilovoltage, kVp) and high-energy images (about 45–49 kVp) using a copper filter instead of a molybdenum/rhodium one. The obtained images are then subtracted according to a specific algorithm and the weighted logarithmic subtracted image is evaluated to detect uptake of iodine contrast agent in enhancing lesions [1–3]. CESM allows visualization of lesions with high vascularity using mammography rather than more expensive technologies such as CT (with iodine-based contrast agents) or MRI (with gadolinium-based contrast agents).

Since the introduction of CESM, several research studies have demonstrated its advantages over conventional MG or MG plus ultrasound (US) [3–5]. Recent studies have demonstrated its sensitivity, specificity, and accuracy to be comparable to breast MRI [6–8]. This study explores quantitative variables in CESM that can be used to distinguish between malignant and benign lesions.

## Material and methods

This retrospective study was accepted by an ethics committee and all enrolled patients provided written informed consent. A total of 167 female patients aged 26–82, with mean age 56 ± 10 years, were included in the study. All subjects underwent spectral mammography CESM performed with Senographe Essential (GE Healthcare CESM). All subjects previously had suspicious lesions found on MG, breast US, or both. Involved patients were diagnostically challenging cases—with dense or inhomogeneous breasts, with suspicion of multicentricity or multifocality in MG/US, and with clinically palpable breast cancers invisible on MG/US or patients with confirmed breast cancer to assess the range of the breast neoplasm.

CESM was performed after intravenous iodine contrast agent administration according to the protocol described in previous publications [4, 9]. Contrast medium was delivered with a power injector, and the first image was obtained 2 min after injection completion. The examination started with the breast not suspected of pathology. In all cases, the craniocaudal (CC) projection of both breasts was obtained prior to the mediolateral-oblique (MLO) projection.

**Fig. 1:**
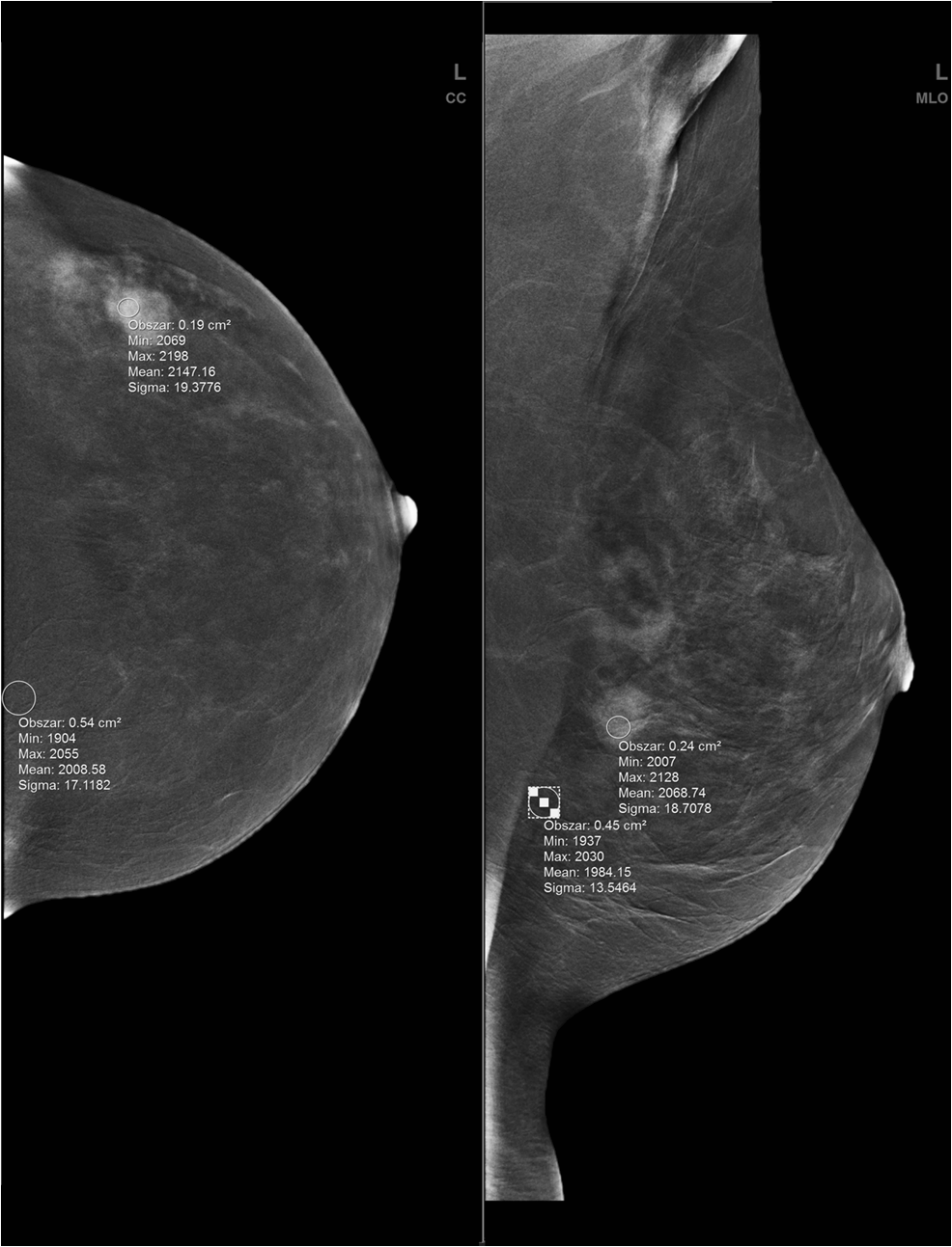
Signal value measurement method on the processed (weighted subtracted) dual-energy image—ROIs placed over the enhancing lesion and background areas in CC (left) and MLO (right) views of the left breast of the same patient

After imaging, the following parameters were evaluated: the number of enhancing lesions in each breast and size and degree of enhancement of each lesion. The localization of each lesion was described in terms of breast quadrant, clock position, and distance from the nipple. Quantitative enhancement was assessed using a region of interest (ROI) placed manually over of most homogenous enhancement area within the lesion. A separate ROI was placed outside the lesion to assess background signal within an area of the most homogenous subcutaneous fatty tissue. ROIs were placed into the fatty tissue to avoid different levels of parenchyma enhancement, to be more representative of the background signal. ROI values were assessed separately for CC and MLO projections (Fig.1). ROI areas were similar within and beyond each lesion, with similarity maintained in both projections. For the background ROI, both mean signal and standard deviation values were recorded. Based on the collected data, percentage signal difference between enhancing lesion and background (% RS) and signal-difference-to-noise ratio (SDNR) were measured for each lesion. Above values were calculated as follows:

$$ {\displaystyle \begin{array}{c}\%\mathrm{RS}=\frac{\overset{\acute{\mkern6mu}}{s_c}-\overset{\acute{\mkern6mu}}{s_b}}{\overset{\acute{\mkern6mu}}{s_b}}\times 100\%\\ {}\mathrm{SDNR}=\frac{\overset{\acute{\mkern6mu}}{s_c}-\overset{\acute{\mkern6mu}}{s_b}}{\sigma_{s_b}}\end{array}} $$

where:

*s’*_*c*_: signal in the lesion,
*s’*_*b*_: signal in the background,
σ: standard deviation.

**Fig. 2:**
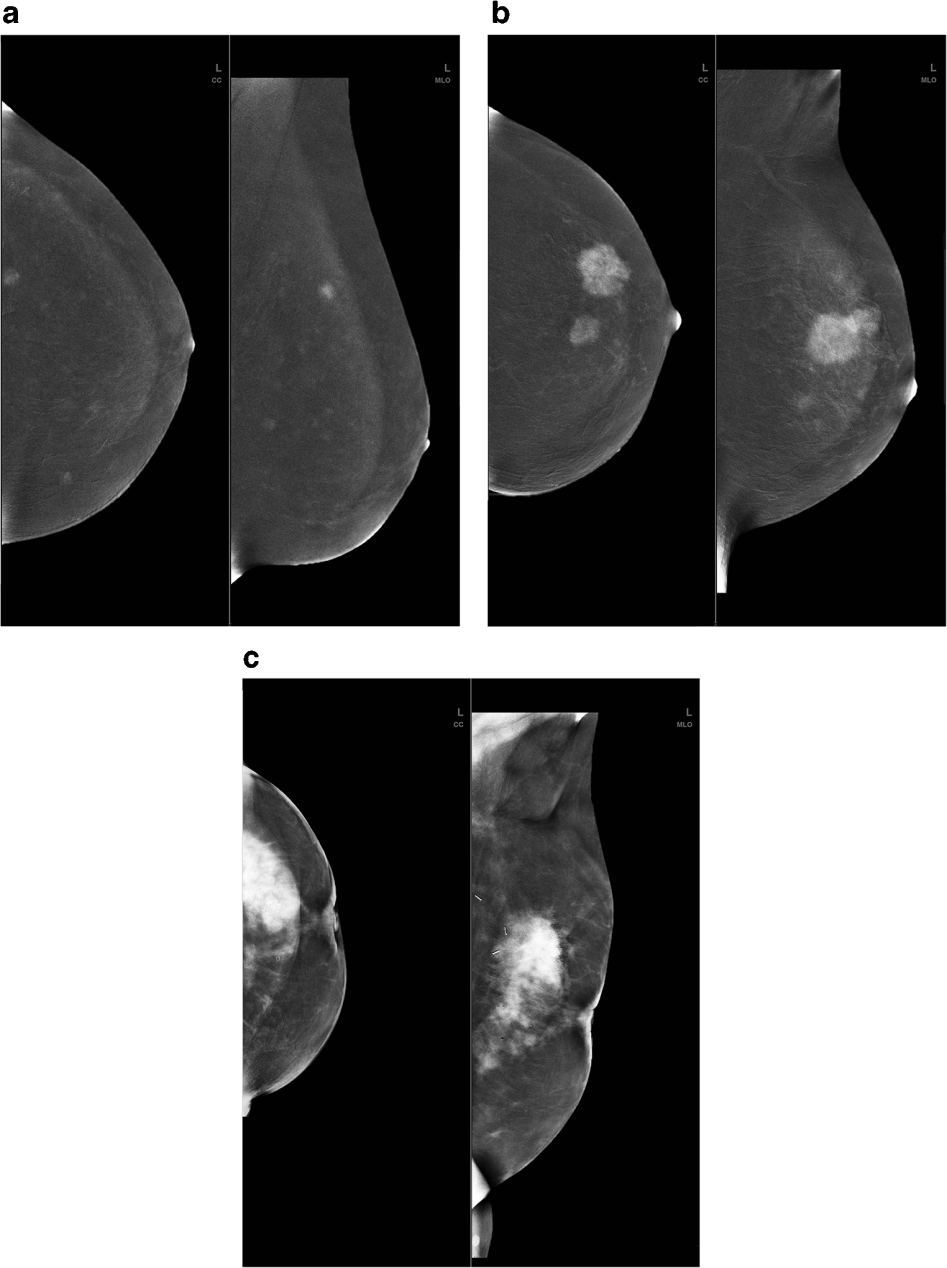
Lesion enhancement levels in CESM processed dual-energy images. **a** Weak enhancement. **b** Medium enhancement. **c** Strong enhancement

After CESM, enrolled subjects underwent further diagnostic examinations including histopathological verification of findings and final determination of the lesion grade. Based on histopathology results, the lesions were divided into three groups: infiltrating cancers, non-infiltrating cancers, and benign lesions. Indices of quantitative enhancement (%RS, SDNR) were evaluated separately for each projection to check if there was a correlation with cancer grade in any lesions. Example lesion enhancement images are presented on Fig.2.

### Statistical methods

Statistical analysis was performed to determine whether %RS and SDNR correlated to histopathological examination results. As the distribution of enhancement indices was not normal, the Kruskal–Wallis test by ranks was used. A significant test (*p* < 0.05) indicates that at least one sample stochastically dominates one other sample. In the first step, the enhancement indices %RS and SDNR calculated for three independent samples were compared: invasive carcinoma, intraductal carcinoma, and benign lesion. The *p* values of differences are shown on the appropriate plots. Next, we determined which sample pairs were significantly different.

Receiver operating characteristic curves (ROC) were prepared and analyzed to determine the diagnostic ability of %RS and SDNR indices. Sensitivity and specificity were calculated for all values of %RS and SNDR, treating them as cut-off points (the value separating malignant from benign). Next, all results were plotted as ROC curves, with sensitivity plotted versus 1-specificity for different cut-off points. Each point on the ROC curve represents a sensitivity/specificity pair corresponding to a particular decision threshold. The area under the ROC curve (AUC) measures how well a parameter can distinguish between two diagnostic groups (malignant versus benign in this case). When the analyzed parameter cannot distinguish between the two groups, the area will be equal to 0.5 (the ROC curve will coincide with the diagonal). When there is a perfect separation between the two groups, the area under the ROC curve equals 1 (the ROC curve will reach the upper left corner of the plot). The AUC is significantly different from 0.5, so there is evidence that the analyzed parameter can distinguish between malignant and benign tissues. Then the optimal cut-off point was obtained, the point on the ROC curve that is the closest to the upper left corner of the plot. The optimal cut-off point can be treated as a diagnostic threshold for best distinguishing between malignant and benign lesions.

### Histopathological examination

Histopathological diagnostics were performed in the pathology department of our hospital for all subjects. The examination was conducted after surgery or core biopsy with each specimen undergoing formalin fixation followed by paraffin embedding. Tumor parameters were assessed by microscopically examining sections stained with hematoxylin and eosin.

## Results

The number of lesions detected in the study population was 195. Within the cohort, 142 (85%) subjects had one lesion, 22 (13%) had two lesions, and 3 (2%) had three lesions. Among all diagnosed lesions, 120 (62%) were assessed to be infiltrating cancers—in this group, 95 (79% of all infiltrating lesions) were invasive ductal carcinomas and 13 were other subtypes; 12 (10% of all infiltrating lesions) were invasive lobular carcinomas; 16 (8%) non-infiltrating cancers; and 59 (30%) were benign. Thirteen (7%) lesions did not enhance in CESM; all non-enhancing lesions were confirmed to be benign under histopathological examination (Table 1). Further analysis involved three main groups: infiltrating, non-infiltrating, and benign lesion. Mean ROI size was 0.39 cm^2^ in CC projection and 0.38 cm^2^ in MLO projection (0.39 cm^2^ overall). ROI values (min, max, and mean for all detected lesions) are presented in Table 2.

**Table 1:** Characteristics of the detected lesions

Lesion type		*N*	%
Invasive carcinoma	Invasive ductal carcinoma	95	49
	Invasive lobular carcinoma	12	6
	Other	13	7
Intraductal carcinoma	Ductal and lobular carcinoma in situ	16	8
Benign lesion	Mixed cases	59	30

**Table 2:** ROI sizes for lesions enhancing on CESM

	*N*	Mean value (cm^2^)	Standard deviation	Min value (cm^2^)	Max value (cm^2^)
CC_ROI	195	0.39	0.30	0.04	3.10
MLO_ROI	195	0.38	0.27	0.04	2.20
Mean_ROI	195	0.39	0.26	0.08	2.40

Analysis of enhancement indices showed that signal values within lesions and signal values within background ROIs were similar in CC and MLO projections. Mean %RS in CC projections was 4% and in MLO projections was 4.7%. Mean SDNR in CC projections was slightly smaller, 6.54, compared with 7.90 in MLO projections. Mean %RS values were correlated with the type of enhancing lesion, infiltrating cancers having the highest values, benign lesions the lowest (Table 3).

**Table 3:** Correlation between %RS and SDNR index vs lesion type and MG projection

	*N*	mean value	Standard deviation (SD)	Min	Max
		%RS_MLO			
Invasive cancer	120	5.5%	3.0%	0.8%	16.6%
Non-infiltrating cancer	16	3.3%	1.9%	1.0%	7.8%
Benign lesions	59	3.3%	2.2%	0.6%	13.7%
		SDNR_MLO			
Infiltrating cancer	120	9.31	5.15	1.34	32.4
Non-infiltrating cancer	16	5.69	3.63	1.07	14.2
Benign lesions	59	5.62	3.74	1.02	22.5
		%RS_CC			
Infiltrating cancer	120	4.8%	3.1%	0.4%	22.8%
Non-infiltrating cancer	16	2.6%	2.0%	0.5%	6.7%
Benign lesions	59	2.6%	1.7%	0.2%	8.1%
		SDNR_CC			
Infiltrating cancer	120	8.44	4.14	2.07	23.9
Non-infiltrating cancer	16	5.21	3.60	1.00	12.8
Benign lesions	59	5.12	3.15	0.92	19.0

This difference between %RS and SNDR for infiltrating cancer and non-infiltrating cancer was statistically significant (*p* < 0.008) and the difference between %RS and SNDR for infiltrating cancer and benign lesions was statistically significant (*p* < 0.0001). Differences between analyzed parameters for benign lesions and non-infiltrating (in situ) cancers were not statistically significant (*p* > 0.8).

**Fig. 3:**
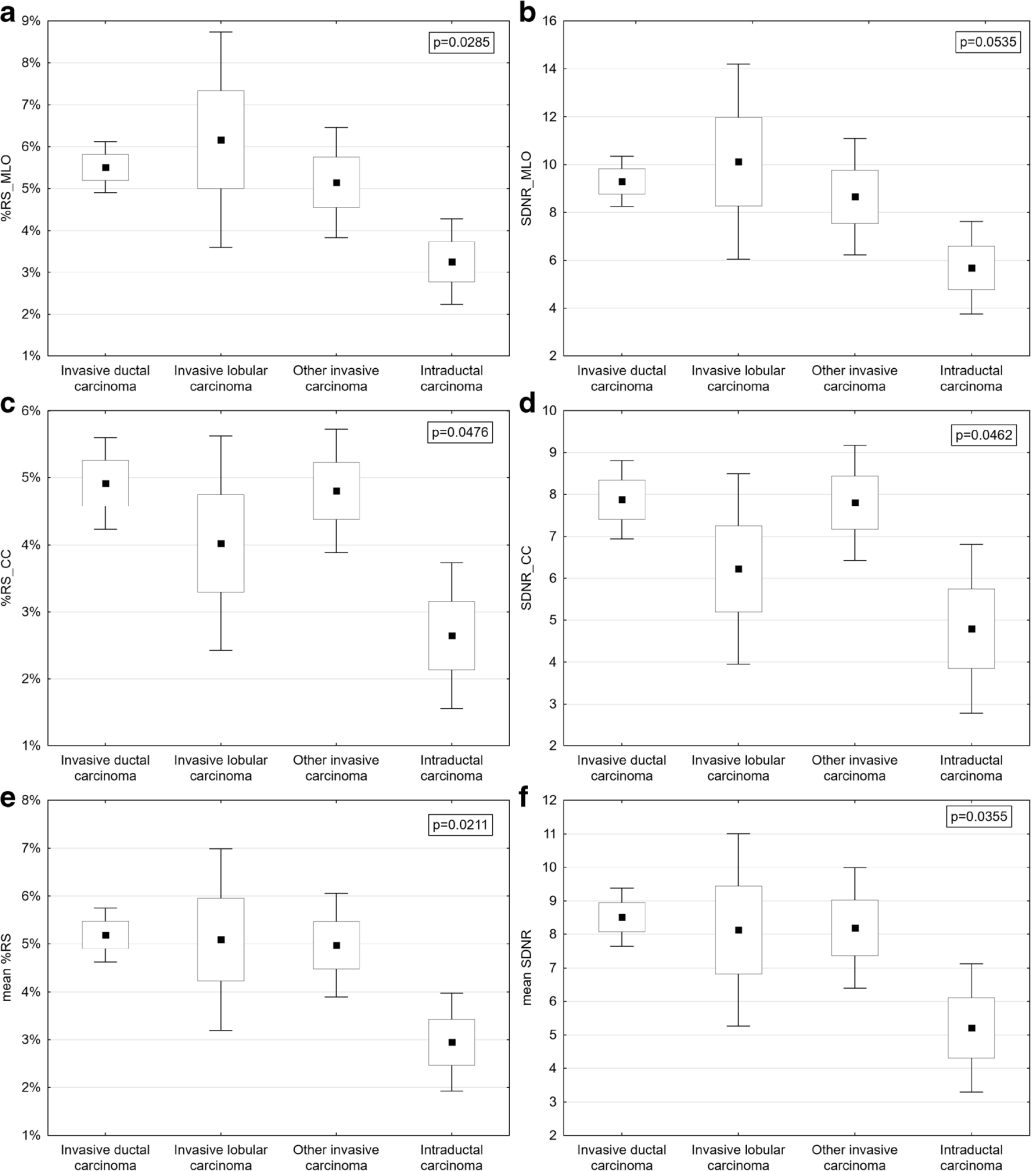
Comparison of %RS and SDNR by cancer status—%RS_MLO vs cancer status (**a**), SNDR_MLO vs cancer status (**b**), %RS_CC vs cancer status (**c**), SNDR_CC vs cancer status (**d**), mean %RS vs cancer status (**e**), mean SNDR vs cancer status (**f**). Differences between parameters are presented with 95% confidence intervals

Correlation between %RS and SDNR and cancer grade depending on projection is also presented in Fig. 3.

Based on ROC analysis, we ascertained that enhancement indices (%RS and SDNR) allowed optimal division of cases into malignant and benign (AUC > 0.5; Tables 4 and 5). We did not determine any significant differences in AUC between index type (%RS, SDNR), result of ROC analysis in case of different projections, and mean values.

**Table 4:** Results of ROC analysis for both projections (CC and MLO) and %RS and SDNR parameters

	Cut-off point	AUC	CI 95%	*p* value
%RC_MLO	3.8%	0.713	[0.64; 0.79]	< 0.0001
%RC_CC	3.4%	0.725	[0.65; 0.80]	< 0.0001
SDNR_MLO	7.222	0.710	[0.63; 0.79]	< 0.0001
SDNR_CC	6.512	0.700	[0.62; 0.78]	< 0.0001

**Table 5:** Clinical performance results for indices of mean enhancement values in both projections CC and MLO

	Sensitivity (%)	Specificity (%)	Accuracy (%)	AUC	CI 95%	*p* value
%RC mean value	49	88	61	0.734	[0.66; 0.81]	< 0.0001
SDNR mean value	49	85	60	0.700	[0.65; 0.80]	< 0.0001
ROI mean value	59	61	60	0.605	[0.52; 0.69]	< 0.0099

**Fig. 4:**
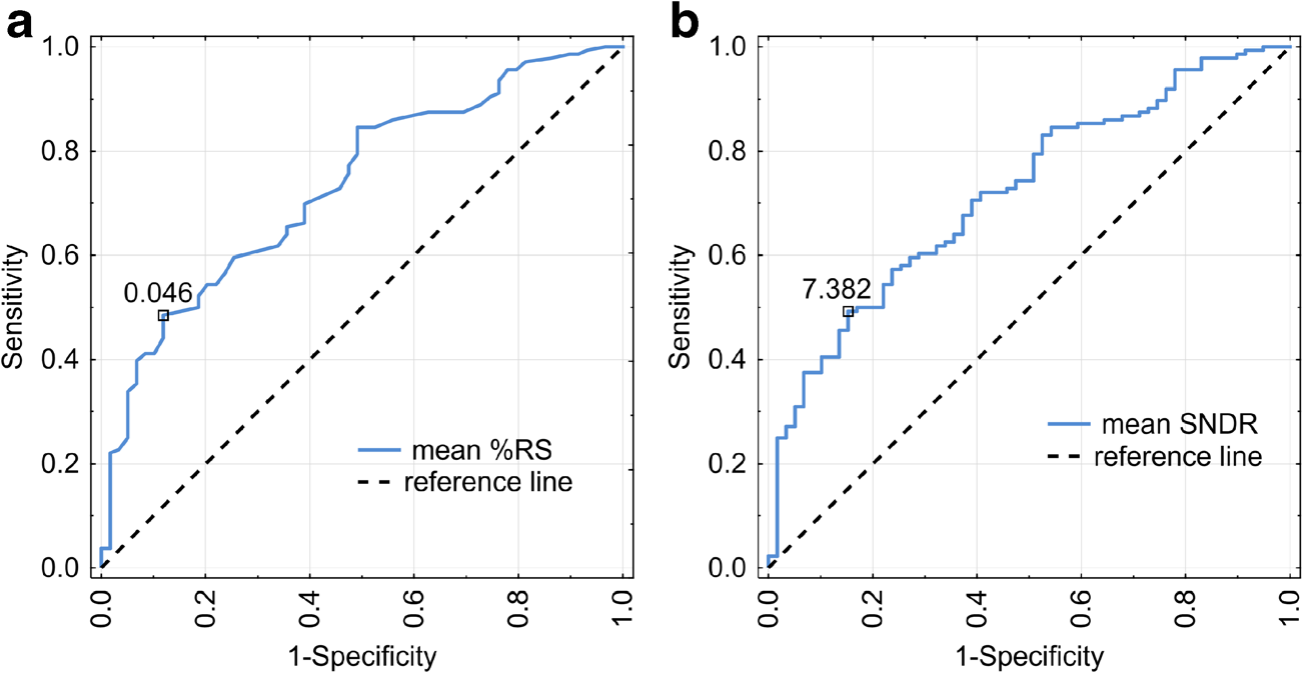
Optimal ROC curves for %RS (solid curve, left) (**a**) and SDNR (solid curve, right) (**b**). Dashed lines show the reference curve (representing the ROC curve for a random distribution of negative and positive test results)

Figure 4 shows ROC curves for mean %RS and SNDR.

Furthermore, we found no statistically significant differences between %RS and SNDR mean values and the lesion type in malignant infiltrating lesions (*p* = 0.96, *p* = 0.93) (Fig. 3).

## Discussion

In this study, preliminary results of quantitative enhancement analysis were presented. We showed that mean values of %RS and SDNR correlate well with lesion malignancy, the highest values of both indices corresponding to infiltrating cancers, the lowest to benign lesions on CESM. This correlation is statistically significant. The difference between benign lesions and non-infiltrating cancers is not statistically significant.

Looking at these results makes it clear that %RS and SDNR are giving essentially the same results. That is because both have the same numerator of signal difference between enhanced lesion and background. %RS is normalized with background signal, while SDNR is normalized with background noise. The better separation of invasive malignant from benign and intraductal lesions suggests that %RS is likely the preferable quantitative analysis parameter.

By analyzing the enhancement values, we determined that focal lesion signal values and background signal values are similar in both CC and MLO projections. The mean value of %RS in CC projection equals 4% and in MLO projection 4.7%. SDNR in CC projection is slightly smaller and equals 6.54 while in MLO 7.9.

Based on ROC analysis, we found that enhancement indices allow for significant separation between invasive breast cancers and benign breast lesions (AUC significantly greater than 0.5). Similar results were achieved in other analyses conducted by Chih-Ying Deng et al [10]: enhancement was also stronger in malignant tumors in comparison with benign ones; ROC characteristics were 0.877, with 95% confidence interval (0.813–0.941). Their results included sensitivity 75.9%, specificity 88.6%, and accuracy 82.3%. Positive likelihood ratio was estimated as 6.681, while negative likelihood ratio as 0.272.

We found little enhancement difference between CC and MLO of the suspicious breast. The difference with Deng’s paper in this respect seems to come from the fact that they imaged CC and MLO of the same breast within a longer time interval.

This study has its limitations. The study cohort included a limited number of patients, especially those having non-invasive cancers. The other limitation is the fact that ROI sizes vary between the lesion and noise sites depending on the homogeneity of pixel values. CESM is a recently developed diagnostic method, so there are only a limited number of studies to which our results can be compared.

## Conclusions

This work has demonstrated a significant correlation between the degree of lesion enhancement in CESM and malignancy—the stronger the enhancement, the higher the probability of malignancy. Quantitative analysis of enhancement levels in CESM can distinguish between invasive cancers and benign or in situ lesions. Further study in this subject is planned to be published.

## Abbreviations

%RS: Percentage signal difference between enhancing lesion and background
AUC: Area under curve
CC: Craniocaudal
CESM: Contrast-enhanced spectral mammography
CI: Confidence interval
MG: Mammography
MLO: Mediolateral
MRI: Magnetic resonance imaging
ROC: Receiver operating characteristic
ROI: Region of interest
SDNR: Signal-difference-to-noise ratio
